# Preparation and Gas Sensing Properties of In_2_O_3_/Au Nanorods for Detection of Volatile Organic Compounds in Exhaled Breath

**DOI:** 10.1038/srep10717

**Published:** 2015-06-01

**Authors:** Ruiqing Xing, Lin Xu, Jian Song, Chunyang Zhou, Qingling Li, Dali Liu, Hong Wei Song

**Affiliations:** 1 State Key Laboratory on Integrated Optoelectronics, College of Electronic Science and Engineering, Jilin University, Changchun, 130012, People’s Republic of China; 2 The State Key Laboratory of Bioelectronics, Southeast University, 210096 P. R. China

## Abstract

A series of In_2_O_3_/Au nanorods (NRs) were fabricated and characterized by scanning electron microscope (SEM), transmission electron microscope (TEM), X–ray diffractometer (XRD) and X–ray photoelectron spectroscopy (XPS). The length to diameter ratios of In_2_O_3_/Au NRs was periodically modulated in the range of 2.9–4.5 through controlling the initial content of indium salt and reaction time. Their gas sensing properties to volatile organic compounds (VOCs) were carefully studied and then applied in exhaled breath detection. The results demonstrate that In_2_O_3_/Au NRs gas sensor can effectively detect acetone at 250 °C and ethanol at 400 °C. The corresponding actual detection limit is as low as 0.1 ppm to acetone and 0.05 ppm to ethanol, respectively. Moreover, by using humidity compensation method, In_2_O_3_/Au NRs gas sensor can clearly distinguish the acetone and ethanol biomarkers in human breath. The main reason of the enhanced gas sensing properties was attributed to the “spillover effects” between Au and In_2_O_3_ NRs. The excellent sensing performance indicates that In_2_O_3_/Au NRs is a promising functional material to actual application in monitoring and detecting diabetes and safe driving area in a noninvasive and more accurate way.

Significant interest has been generated in the field of selective detection of specific VOCs which also called biomarkers in exhaled breath from the metabolism process^1^,^2^,^3^,^4^,^5^,^6^. What’s important, these biomarkers can intuitive indicate some abnormalities of human body, including problems with carbohydrate digestion and blood ethanol levels^7^,^8^,^9^. For example, the routine method to detect diabetes were usually monitoring the glucose concentration in patient’s blood; however, diabetes can also be reflected by a biomarker of gaseous acetone in human breath, and clinical data showed that the exhaled acetone of diabetes exceeded 1.8 ppm, while for healthy people was only 0.3–0.9 ppm^10^,^11^,^12^. Obviously, direct analyzing the exhaled breath possesses advantages of noninvasiveness, ease of operation, and accuracy. Thus, despite the wide range of constituents in human breath may interfere the actual analysis and make it complicated, specific biomarkers are still intensively studied.

Recently, nanomaterials based sensors are considered to be a promising clinical and laboratory diagnostic tool, because its large surface–to–volume ratio, controllable structure, easily tailored chemical and physical properties, which bring high sensitivity, fast dynamic process, and even the increasing specificity^13^,^14^,^15^. Among various nanomaterials, metal oxide semiconductor based chemiresistive sensors are likely to become a portable real–time breath detector, because of the small size, low cost, ease of operation, and particularly strong correlations with instrumental analysis. Although there is no metal oxide based sensors being developed for human breath sensing, some related works have been reported, Righettoni *et al*. fabricated Si doped WO_3_ sensors for quantitative analysis of acetone in dry and 90% relative humidity (RH) air samples^16^. Very recently, Shin *et al*. prepared Pt nanoparticles (NPs) functionalized SnO_2_ fibers through electrospinning which could be a potential material for the detection of gaseous acetone and toluene, which are the biomarkers for the diabetes and lung cancer, respectively^12^. Inyawilert *et al*. synthesized In_2_O_3_ sensing film by a sparking process which can use to detect ethanol and acetone^17^. However, the working temperature towards different gas as mentioned above could not be clearly distinguished. Nevertheless, the performances, including sensitivity, selectivity, and response time, and else, of conventional gas sensors generally could not satisfy the requirements for breath analysis because the gaseous biomarkers in exhaled breath were complex in contents and with low concentrations. Therefore, the reports of sensor focused on the practical detection of biomarkers in expired air or even in simulated exhaled breath environment were rare. Beyond that, no instance demonstration about dual mode sensor that can diagnose two different biomarkers towards exhale breath has been reported to the best of our knowledge.

One–dimensional (1D) metal oxide nanostructures have been identified to be one of the most effective nanoarchitectures for chemiresistive VOCs detection^18^,^19^,^20^,^21^,^22^,^23^,^24^. Especially, the dual mode sensing properties can be obtained or optimized in 1D system due to their high gas accessibility and large specific surface area^23^,^25^,^26^. Accordingly, in this paper, we present a facile, inexpensive and one–step method to fabricate the In_2_O_3_/Au NRs through co–precipitation method. By this way, the small Au NPs were effectively embedded in In_2_O_3_ NRs and acted as sensitizer. In particular, In_2_O_3_/Au gas sensor not only can detect acetone lower to 10 ppb at 250 °C, but also can detect ethanol lower to 50 ppb at 400 °C. Moreover, this sensor showed good anti–interference to humidity and could effectively detect gaseous acetone and ethanol in simulated exhale breath environment. And then, a humidity compensation method was used to increase the detection accuracy and the clinic test was also conducted.

## Results and discussions

### Morphological and Structural Characteristics

**Figure 1:**
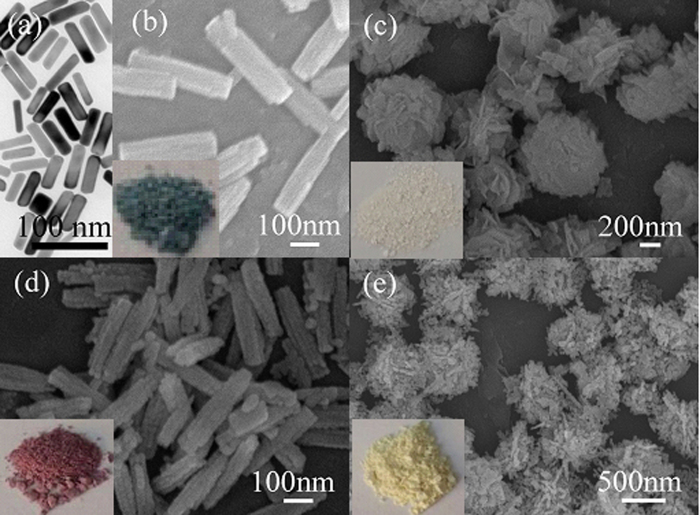
(**a**) TEM of Au NRs used in this work. SEM images of (**b**) S3 In_2_O_3_/Au NRs precursor sample and (**c**) pure In_2_O_3_ precursor sample, which both have 0.2 g initial In(NO_3_)_3_ and obtained at the reaction time of 120 min, (**d**) S3 In_2_O_3_/Au NRs and (**e**) pure In_2_O_3_ samples after annealing. The insets in (**b–e**) are the pictures corresponding to each sample.

The morphologies and nanostructures of the precursor and annealing samples are first illuminated. The uniform Au NRs used in this work are as shown in Fig. 1a, which are ~15 nm in diameter and ~50 nm in length. Figure 1b shows the In_2_O_3_/Au precursor sample (S3, atom ratio of Au/In = 1.8%) contained with 0.2 g In(NO_3_)_3_ and Au NRs (with reaction time of 120 min), and the as prepared precursor exhibits uniform NRs structures and small Au NRs are not observed elsewhere. Well, unlike with In_2_O_3_/Au precursor, the pure In_2_O_3_ without addition of Au NRs formed flower–like microspheres instead of NRs (Fig. 1c). As in a hydrothermal environment, urea played a crucial role in the formation of this flower–like precursor which could hydrolyze and release OH^−^ and CO_3_^2 − ^27. The nucleation and growth of In(OH)CO_3_ thus stemmed from the precipitation of In^3+^ by OH^−^ and CO_3_^2−^ ions. Then the small In(OH)CO_3_ NPs aggregated due to the high surface energy of the NPs and finally self–assembled to flower like spheres^28^. After addition of Au NRs which was positively charged because of residual CTAB on its surface, the negatively charged OH^−^ and CO_3_^2−^ ions tended to gather around Au NRs, and then induced the heterocoagulation process start from the surface of Au NRs and finally self–assembled to NR structure, that is to say, Au NRs played a role as template in forming the In_2_O_3_/Au NRs^29^. After annealing, the corresponding morphologies are maintained and have no significant change except the size shrank (Fig. 1d,e). The In_2_O_3_/Au NRs after annealing have the average diameter of 75 nm and length of 310 nm, that is to say, the length to diameter ratio is about 4.1. Besides, the color of different samples as mentioned above is very different. As can be clearly seen in inset of Fig. 1b–e, the color of In_2_O_3_/Au precursor is dark green, while the color of pure In_2_O_3_ is milk white to the naked eye, after annealing the color of In_2_O_3_/Au NRs is dark red, while the pure In_2_O_3_ is light yellow. This indicated that Au NRs were successfully introduced into In_2_O_3_/Au NRs.

Besides, the In_2_O_3_/Au NRs samples with different initial amount of In(NO_3_)_3_ and reaction time were also synthesized. As shown in Figure S1, the different initial amount of In(NO_3_)_3_ and reaction time have a little influence on the morphologies. The length to diameter ratios are about 3.6, 3.8, and 4.5, respectively to 0.1g (Figure S1a, S1, atom ratio of Au/In = 3.6%), 0.15 g (Figure S1b, S2, atom ratio of Au/In = 2.4%), and 0.3 g (Figure S1c, S4, atom ratio of Au/In = 1.2%) In(NO_3_)_3_ at the reaction time of 120 min, which show a slight increase trend with the increasing of the In(NO_3_)_3_ amount. When the reaction time is different, the length to diameter ratios is changed. As can be seen from Figure S1d, when the reaction time is 50 min (S5), the length to diameter ratios is about 2.9, which is smaller than the sample with the reaction time of 120 min. While when the reaction time is 180 min (S6) the length to diameter ratios is nearly same with 120 min, as can be seen from Figure S1e.

**Figure 2:**
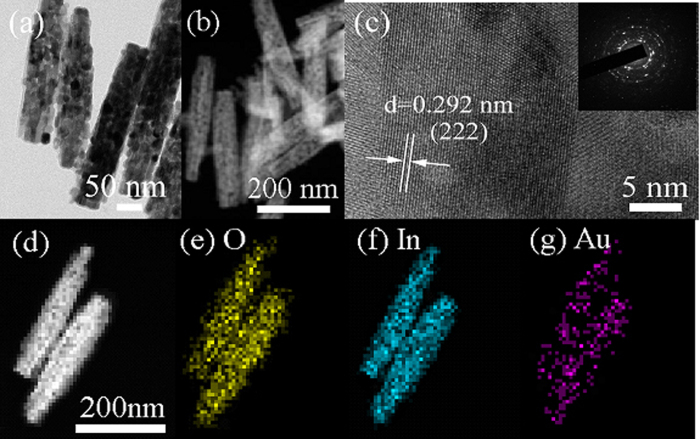
(**a**) FETEM, (**b**) STEM, (**c**) HRTEM image, and SAED (inset of **c**) of S3 In_2_O_3_/Au NRs sample after annealing. (**e–g**) EDX elemental mapping images of O, In, and Au of S3 In_2_O_3_/Au NRs after annealed.

The detail information about the microstructure and morphology of the as–synthesized In_2_O_3_/Au NRs sample was further carried out by TEM and STEM. It can be seen that the In_2_O_3_/Au NRs contains a lot of interspaces, indicating a large surface area as shown in Fig. 2a,b. Moreover, the clear lattice fringes can be observed in entire HRTEM image revealed that the nanostructure is consisted of nanosize grain particles (Fig. 2c) and the lattice fringes is d = 0.292 nm, which match well with the crystallographic planes of cubic In_2_O_3_. The corresponding SAED pattern (inset of Fig. 2c) indicated that the nanostructure of the as–synthesized In_2_O_3_/Au NRs is polycrystalline, being in agreement with the TEM results.

EDX mappings were carried out to further investigate the specific distribution of In and Au elements in In_2_O_3_/Au NRs samples after annealing as shown in Fig. 2. Homogeneous distribution of In and O elements can be clearly seen, and exhibit the NRs structure. For Au element, the corresponding distribution is uniform dispersion in In_2_O_3_/Au NRs rather than accumulated as small Au NRs structure, that is to say, Au NRs are melting then uniformly dispersed in the In_2_O_3_ NRs after annealing, which is consistent with our previous study^29^.

**Figure 3:**
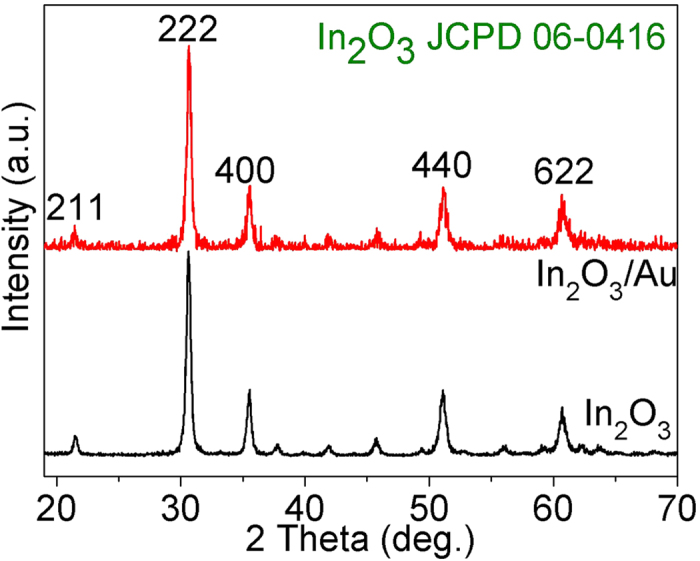
XRD patterns of S3 In_2_O_3_/Au NRs as well as pure In_2_O_3_.

The typical XRD patterns of the as–synthesized In_2_O_3_/Au NRs as well as In_2_O_3_ after annealing were shown in Fig. 3. Pure cubic phase (JCPDS card no. 06–0416) can be well detected in both In_2_O_3_/Au NRs and In_2_O_3_ samples and high intensity of the diffraction peaks in the XRD pattern indicate that the samples have high crystallinity. Besides, in In_2_O_3_/Au NRs samples, the diffraction peaks of Au are not obvious due to the small doses of added Au NRs.

### XPS

**Figure 4:**
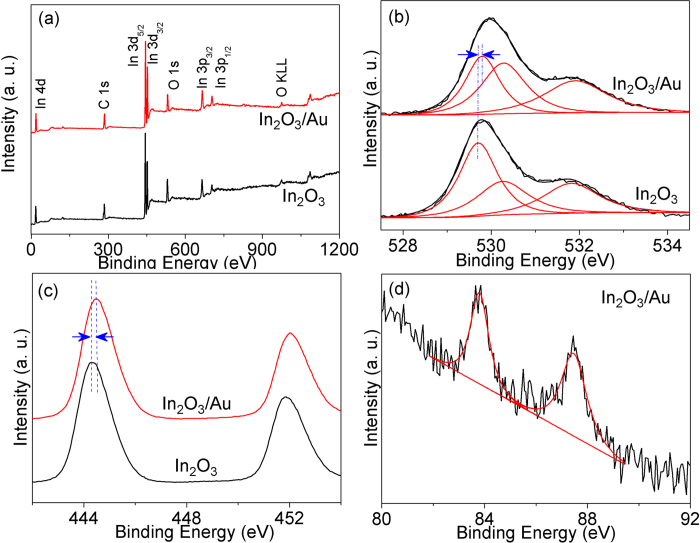
(**a**) Survey, (**b**) O1s, (**c**) In 3d high resolution XPS spectra of S3 In_2_O_3_/Au NRs and pure In_2_O_3_. (**d**) Au 4f high resolution XPS spectrum of S3 In_2_O_3_/Au NRs

In order to obtain a more detailed chemical composition of the as–prepared In_2_O_3_/Au NRs, the XPS spectra were conducted and compared with that of the pure In_2_O_3_.The complete spectra of the samples are shown in Fig. 4a, which confirm the presence of In, O, and C atoms in In_2_O_3_/Au NRs as well as pure In_2_O_3_.While, because the content of Au is relatively small, the XPS peaks of Au are not obvious in the complete spectrum of In_2_O_3_**/**Au NRs^30^. The detailO1s XPS spectra of In_2_O_3_/Au NRs and pure In_2_O_3_ samples are enlarged in Fig. 4b. As is shown, both the O1s XPS spectra of In_2_O_3_/Au NRs and In_2_O_3_ samples display three peaks. In detail, the binding energies around 530.3 eV and 531.9 eV are assigned to the deficient oxygen and the adsorbed OH groups or adsorbed oxygen species, respectively^31^. Obviously, it can be seen that the content of deficient oxygen in In_2_O_3_/Au NRs are much higher than that of pure In_2_O_3_. As is calculated, the corresponding deficient oxygen atomic ratio percentages are 35.1% and 23.4% in In_2_O_3_/Au NRs and pure In_2_O_3_, respectively, suggesting the more surface oxygen vacancies were formed in In_2_O_3_/Au NRs. The main O1s XPS peak at 529.7 eV in In_2_O_3_/Au NRs corresponds to the lattice oxygen of crystalline In_2_O_3_, and compared to pure In_2_O_3,_ a chemical shift to higher binding energy side is observed (529.8 eV in pure In_2_O_3_). The same phenomenon can be observed in the characteristic spin–orbit split XPS data of trivalent indium as shown in Fig. 4c. The binding energies of In 3d_5/2_ and In 3d_3/2_ are at 444.5 and 452.0 eV, respectively. Compared with the pure In_2_O_3_, clearly shift to the high binding energy also can be seen, those shifts may relate to the different local environment due to the introduced of Au. Moreover, typical XPS peaks of the core level region of Au4f can be observed in as–prepared In_2_O_3_/Au NRs. As can be seen in Fig. 4d, Au is present only in the Au^0^ state^32^,^33^,^34^. This indicates the existing of Au in In_2_O_3_/Au NRs, which is consist with above analysis.

### The Enhanced Gas Sensing Properties towards VOCs

**Figure 5:**
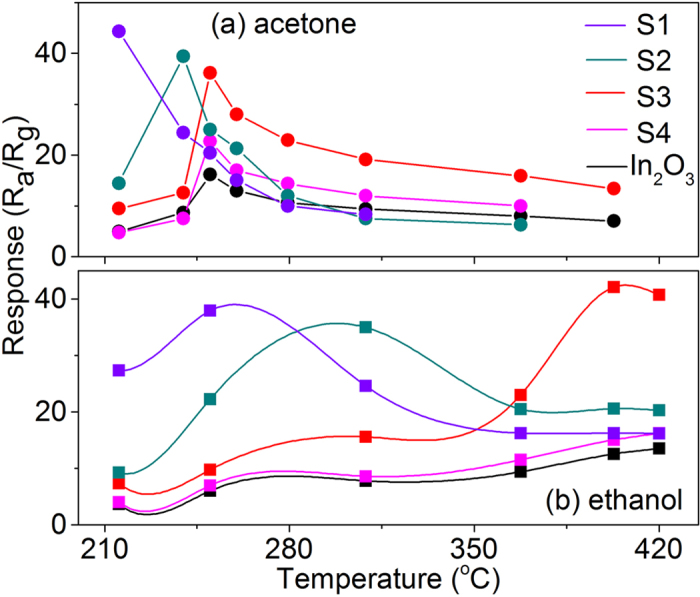
Dual mode responses of S1–S4 In_2_O_3_/Au NRs gas sensors compared with pure In_2_O_3_ gas sensor for 50 ppm acetone and ethanol as a function of operating temperature.

The gas sensing properties of the In_2_O_3_/Au NRs as well as pure In_2_O_3_ were carefully studied and compared, and then exhaled gas environment was simulated to further evaluate the performance of as prepared sensors towards to practical application. First, the responses of the In_2_O_3_/Au NRs with different initial amount of In(NO_3_)_3_ and reaction time compared with pure In_2_O_3_ sensors to 50 ppm acetone and ethanol gases as a function of operating temperature are exhibited in Fig. 5. It can be seen that the optimum temperature and effective response of the gas sensors are both influenced by the atom ratio of Au/In. Note that the different reaction time has little affluence on the sensing behavior (S5 and S6, response curves are not shown), including working temperature and response value, due to the similar Au/In and length to diameter ratios value. For acetone gas, all the sensors exhibit a good response to acetone in the lower temperature range. Except S1 gas sensors, the responses first increase to their optimal working temperature and then decreases as the temperature further increases. The optimal working temperature and corresponding responses for S1–S4 In_2_O_3_/Au NRs gas sensors are (215 ^o^C, 44.3), (240 ^o^C, 39.4), (250 ^o^C, 36.2), and (250 ^o^C, 22.7), respectively. Compared to pure In_2_O_3_ gas sensor (250 ^o^C, 16.2), the response improves gradually when the atom ratios of Au/In increases (from S4 to S1), while the optimal working temperature shows a decreased trend only when the atom ratio of Au/In > 3.6. For ethanol gas, compared to pure In_2_O_3_ gas sensor (400 ^o^C, 12.6), the optimal working temperature and corresponding responses for S1–S4 In_2_O_3_/Au NRs gas sensors are (250 ^o^C, 38.0), (310 ^o^C, 35.0), (400 ^o^C, 42.1), and (400 ^o^C, 15.1), respectively. As can be seen, the optimal responses mainly appear in the relatively higher temperature range and the changing trend of working temperature is similar with that of acetone gas. While the change of response value has only a little difference when the atom ratio of Au/In is larger than 3.6.

Furthermore, the response times and recovery times of different sensors working at corresponding optimal temperature were also evaluated in Table S1. As is listed, for acetone gas, the response time and recovery time show decreased trend when the introduced Au amount increases at first (In_2_O_3_, S4, and S3, respectively). However, when the Au amount further increases (S2 and S1), with the decrease of corresponding optimal working temperature, the response time and recovery time become longer rapidly. The changing trend of response time and recovery time for the ethanol gas is similar to that for acetone; however, the changing range is relatively small in the higher temperature range in the case of ethanol. The influence mechanism of the introduced Au on the sensing performance of the studied sensors is carefully discussed later in this article. It is worth mentioning that from the view of response, temperature difference, response and recovery time to this two gases, the as studied sensors, especially the S3 In_2_O_3_/Au NRs sensor, have dual mode sensing properties, which can quickly detect acetone gas at a relatively low working temperature (250 °C in our case) and detect ethanol at a higher temperature section (400 °C in our case), and the response of S3 In_2_O_3_/Au NRs sensor is ~2.2 and 3.3 times higher than that of pure In_2_O_3_ sensor under optimal working temperature, respectively.

**Figure 6:**
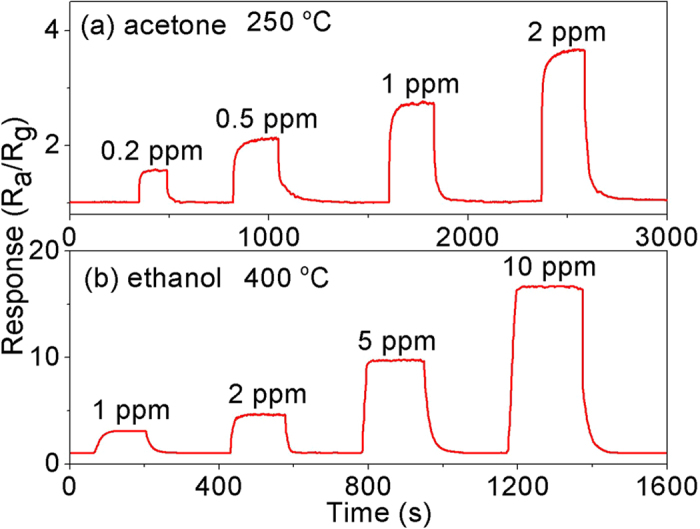
Dynamic responses of S3 In_2_O_3_/Au NRs gas sensor to different acetone concentrations (0.1–2 ppm) at 250 °C and ethanol concentrations (0.05–10 ppm) at 400 °C.

Generally, the biomarker gases in exhaled breath are low in concentrations. In order to obtain more information about the dual mode properties of In_2_O_3_/Au NRs sensor toward to exhale breath, detail tests were conducted focused on a very low gas concentration which can meet the requirement of exhaled gas detection. First, the dynamic responses to different concentration of acetone at 250 °C and ethanol at 400 °C were investigated. Four response and recovery curves are shown in Fig. 6 corresponding to 0.2, 0.5, 1 and 2 ppm acetone gas and 1, 2, 5 and 10 ppm ethanol gas, respectively. As depicted, the sensors both exhibit sharply rise when acetone or ethanol gas were injected, while quickly drop to the initial state when exposed to air, which is consistent with the sensing mechanism of n–type semiconductors.

**Figure 7:**
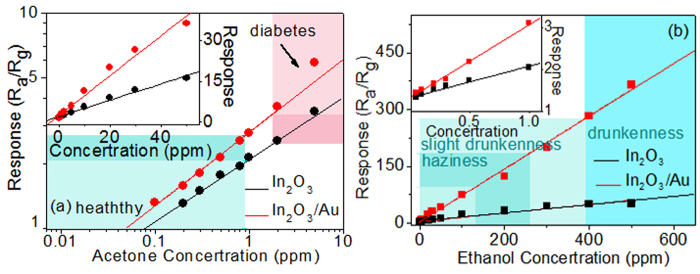
S3 In_2_O_3_/Au NRs and pure In_2_O_3_ gas sensors response curves to different (**a**) acetone concentrations (0.1–50 ppm) at 250 °C and (**b**) ethanol concentrations (0.05–650 ppm) at 400 °C.The dots are experimental data and the straight lines are the linear fitting functions.

Figure 7 shows the responses of In_2_O_3_/Au NRs and pure In_2_O_3_ gas sensors versus different acetone and ethanol concentrations at 250 °C and 400 °C respectively. As can be seen, the sensors show a good and wide linear relationship with the growth of the acetone and ethanol concentrations in all the studied range. Besides, the In_2_O_3_/Au NRs sensor exhibits good resolution to low concentration gas. For acetone detection, the In_2_O_3_/Au sensor response is below 2.5 for healthy humans (<0.9 ppm) and above 3.7 for diabetics (>1.8 ppm). This 48% response increase may allow reliable diagnosis of diabetic patients by breath acetone monitoring. For ethanol detection, the large linear range clearly covered the different alcohol levels encountered in breath (130–260 ppm, 260–390 ppm, and 390–650 ppm in breath corresponding to haziness, slight drunkenness, drunkenness, respectively) after drinking^35^. Furthermore, it can actually detect as low as 0.1 ppm acetone gas with a response of 1.3 at 250 °C and as low as 0.05 ppm ethanol gas with a response of 1.4 at 400 °C. These concentrations are much lower than the actual detection limit of diabetes (1.8 ppm), permissible limit level for driving under the influence of alcohol (78 ppm), and even detection limit of ethanol selective detection limit for the human sense of smell (6.1 ppm)^12^,^32^. Moreover, for acetone detection, the low detection limit of acetone is calculated to be 90 ppb for In_2_O_3_/Au NRs sensor when R_a_/R_g_ ≥ 1.2 was used as the criterion for reliable gas sensing, confirm that the as–fabricated gas sensor is a promising candidate for the detection low concentration of acetone and ethanol biomarkers in breath with a portable metal oxide detector.

In addition, the longtime stability of the In_2_O_3_/Au NRs compared with pure In_2_O_3_ gas sensors to 1 ppm acetone and 5 ppm ethanol were measured at 250 °C and 400 °C respectively as shown in Figure S2. As is depicted, after 30 days, the response of In_2_O_3_/Au NRs sensor only decreased about 4% for acetone and 5% for ethanol in comparison to the original values, whereas the response of pure In_2_O_3_ sensor deceased about 12% and 13% to acetone and ethanol, respectively. This demonstrates that the sensor based on the In_2_O_3_/Au possesses excellent long time stability.

### The Dynamic Process of the Sensors Towards VOCs

Besides, the response times and recovery times *vs*. different acetone and ethanol concentration of pure In_2_O_3_ and S3 In_2_O_3_/Au NRs gas sensors are listed in Table S2. As is calculated, the average response times and recovery times of S3 In_2_O_3_/Au NRs gas sensor are 10.8 s and 18.4 s for acetone gas and 9 s and 13.4 s for ethanol gas, respectively, which are systemically shorter than that of pure In_2_O_3_ gas sensor (14.8 s and 26.2 s for acetone gas and 9.4 s and 17.8 s for ethanol gas, respectively). This can be attributed to the introduction of the Au NPs which have catalytic properties. In addition, the dynamic processes of ethanol of both sensors are faster than that of acetone, this is because the higher the working temperature the faster the reaction of thermodynamics.

**Figure 8:**
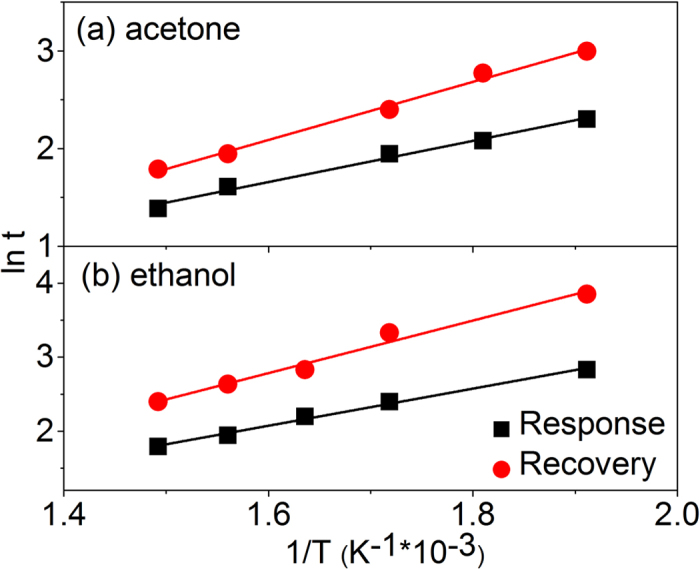
Logarithm of the response and recovery time (both in s) constant versus the reverse of temperature for (**a**) acetone and (**b**) ethanol. The dots are experimental data of S3 In_2_O_3_/Au NRs and the lines are the linear fitting functions.

Actually, we have measured the response and recovery time constants at various temperatures to acetone and ethanol, respectively. As is known, the response and recovery is a reversible process. No matter in which process, a certain potential barrier should be overcame to complete the reaction. According to our previously work^36^, the response (*τ*_*res*_) and recovery time (*τ*_*rec*_) constants can be written as a function of temperature according to the well–known thermal activation function:
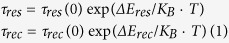
where *τ*_*res*_(0) and *τ*_*rec*_(0) are time coefficients, which depend on surface to volume ratio and reaction mechanism. *K*_*B*_ is the Boltzmann’s constant and *T* is the absolute temperature. Δ*E*_*res*_ and ΔE_*rec*_ present the forward and backward reaction barrier heights, respectively, while τ_*res*_ and τ_*rec*_ present the forward and backward reaction time. Figure 8 show the Napierian logarithm form of dynamic time constant as a function of the reverse of temperature for acetone and ethanol gases, respectively. It can be seen that the response and recovery time constants both increase linearly with the decrease of temperature. By fitting, ΔE_*res*_ and ΔE_*rec*_ are deduced to be 211 and 298 meV for acetone gas, and 251 and 355 meV for ethanol gas. As is calculated, the forward reaction barrier heights always lower than the backward reaction barrier heights, this indicated that to complete recovery processes need to overcome higher barrier height which may lead to the recovery times always longer than the response times in our case. Besides, the reaction barrier heights of ethanol gas are systematically higher than that of acetone, that is to say, more energy is needed to completed the dynamic processes in ethanol environment, this may have some relation with why the optimum operation temperature of the sensors to ethanol gas is higher than that to acetone gas.

### Selectivity and Humidity Effect in Simulated and Human Exhaled Gas Environment

**Figure 9:**
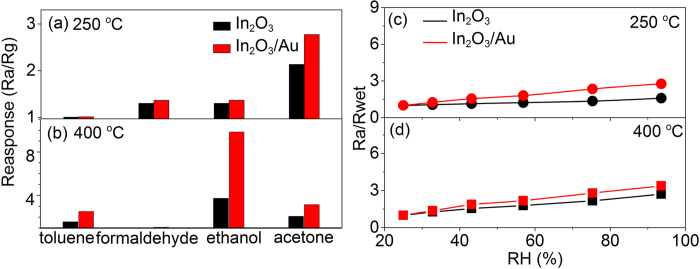
Selective tests of S3 In_2_O_3_/Au NRs compared with pure In_2_O_3_ gas sensors for (**a**) 1 ppm toluene, ethanol, and formaldehyde at 250 °C and (**b**) 5 ppm formaldehyde, toluene, and acetone at 400 °C. Ra/Rwet responses of S3 In_2_O_3_/Au NRs and pure In_2_O_3_ gas sensors versus RH ambience at (**c**) 250 °C and (**d**) at 400 °C.

The constituent of people exhale breath gas is complicated, which composed by CO_2_, O_2_, some other VOCs and biomarker gases, and especially high humidity levels (>80% RH at 1 atm and 25 °C). Thus, the selectivity and the response of the humidity environment are important in the actual breath gas measurement. Here, selectivity of In_2_O_3_/Au NRs sensor were evaluated at 250 °C and 400 °C respectively towards to some typical biomarkers (toluene to lung cancer, formaldehyde to cardiovascular disease) in exhale breath and compared with pure In_2_O_3_ sensor (acetone gas concentration was 1 ppm and ethanol was 5 ppm)^12^,^37^,^38^. As demonstrated in Fig. 9a,b, both two kinds of sensors possess good selectivity to detect acetone at 250 °C and ethanol gas at 400 °C, especially the In_2_O_3_/Au NRs sensor, which shows very low response or almost insensitive to the other typical interference gases at the same temperature.

The interference of humidity is an important problem that must consider in breath sensing, because the VOCs signals could be screened by the high humidity levels in breath, and the small fluctuations in humidity also have big effect on the sensitivity of VOCs^39^. Here, the influence of the high humidity level was considered in our research. Figure 9c,d shows the curve of R_a_/R_wet_ response where R_wet_ is the resistance of sensor at a given RH environment versus RH ambience. Usually, R_wet_ is interpreted as the interaction between the surface adsorbed oxygen and the water molecules which induces a decrease in baseline resistance of the gas sensor. It can be seen that R_a_/R_wet_ value of both In_2_O_3_/Au NRs and pure In_2_O_3_ sensors rise slightly in the as studied RH range (25%–94%), In the studied RH range, the R_a_/R_wet_ value of In_2_O_3_/Au sensor only changed from 1.0 to 2.8 at 250 °C and 1.0 to 3.4 at 400 °C compared to pure In_2_O_3_ sensor that changed from 1.0 to 1.6 at 250 °C and 1.0 to 2.7 at 400 °C. The In_2_O_3_/Au sensor shows a little larger affected than pure In_2_O_3_ sensor due to the sensitized effect of Au.

**Figure 10:**
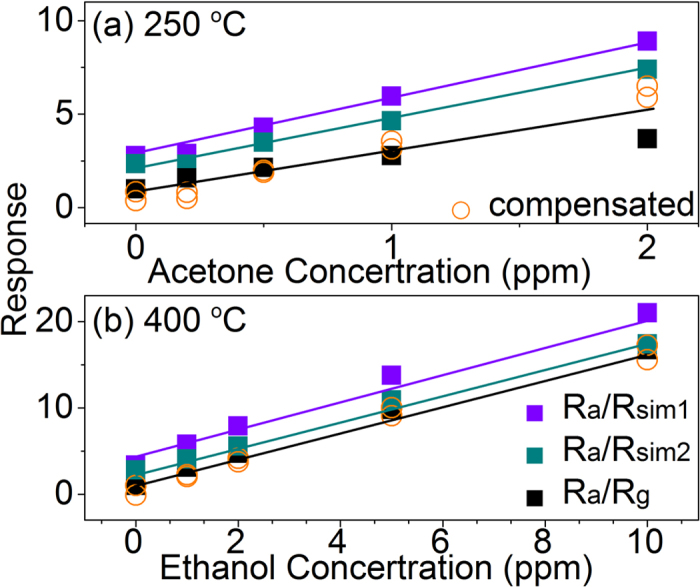
S3 In_2_O_3_/Au NRs gas sensor response curves to different (**a**) acetone concentrations (0.2–2 ppm) at 250 °C and (**b**) ethanol concentrations (1–10 ppm) at 400 °C under simulate gas atmosphere. sim1:93.5 ± 1% RH and sim2:75.5 ± 1% RH. The open circles are the compensated data.

Since the as prepared In_2_O_3_/Au sensor exhibits good dual mode sensing properties towards to very low acetone and ethanol concentration as studied above, the response in simulated exhaled gas environment was further investigated and compared, as shown in Fig. 10. In this work, in order to meet the requirement of the volume fraction of the main components gas is 78% for N_2_, 17% for O_2_, 4% for CO_2_, and 1% for other gas, the simulated exhaled gas environment was built by mixed ambient atmosphere with a certain amount of CO_2_ and different concentration of acetone (250 °C) or ethanol gas (400 °C) in high RH surroundings, moreover, take into consideration the high humidity and individual difference in exhaled gas environment, 93.5 ± 1% RH (sim1) and 75.5 ± 1% RH (sim2) conditions were also provided.

As depicted in Fig. 10, compared to the response in atmospheric air (R_a_/R_g_), the responses in simulated exhaled gas environment (R_a_/R_sim_) of S3 In_2_O_3_/Au NRs sensor shows gradually increased trend with the increasing of the RH value, because the sensor almost has no response to additional CO_2_, the main reason can be attributed to the effect of high RH conditions. Besides, all the R_a_/R_sim_ responses in simulated exhaled gas environment of In_2_O_3_/Au NRs sensor show good linearity on the whole, and the slops of the three curves of acetone at 250 °C and ethanol at 400 °C are independent of the RH level, indicating the response to the VOCs is not effected by the background humidity. In this case, a humidity compensation method could use to increase the accuracy of the sensor response in the environment of high humidity levels or fluctuations in humidity, which has been proved to be effective^40^. In this method, the humidity compensation parameter can be extracted by deducting the humidity background value when there is no target VOCs based on the estimated sensor response in Fig. 10. Note that because the ambient humidity is 25% RH in our test environment, all the response signals are compensated to 25% RH level. As can be seen, after applying compensation, the relative effect of humidity can be counteracted (expressed by the open circle in Fig. 10), and the responses to a specific VOCs concentration are mainly located at the same place, indicating the effect of humidity variations could be effectively eliminated through this humidity compensation method in our case.

**Figure 11:**
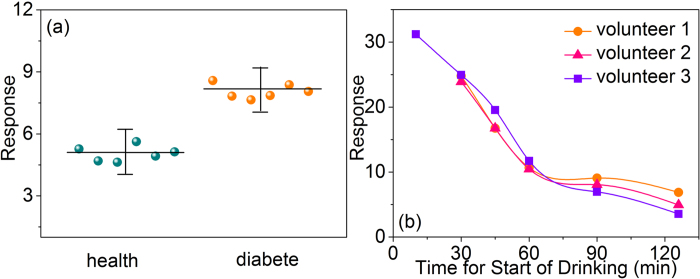
The compensated clinic test data to (**a**) diabetic and healthy volunteers, (**b**) ethanol response curves after drinking white wine of three healthy volunteers.

Furthermore, the clinic test were conducted to test the sensing properties of S3 In_2_O_3_/Au NRs sensor to the VOCs in human breath and the RH compensation method also was applied. For acetone detection, 1 diabetic and 6 healthy volunteers participated in the test. As shown in Fig. 11, the response data were obtained by sampling one time for each healthy volunteer and six times for diabetes volunteer. The results show that the sensor response is about 8. of diabetic, while 5.1 of healthy person. All the response values are little higher compared to that in simulation environment; the reason can be attributed to the complex environment in exhaled breath. For ethanol detection, the corresponding response vales were obtained by sampling the breath of 3 healthy volunteers who have drink white wine (42% vol) in amounts calculated to provide 0.15–0.20 g white wine per kg body weight. As exhibited in Fig. 11b, the volunteers started to drink the white wine at zero point, and we continuous monitoring the variation of sensor response up to 125 min, in the whole range, the responses showed a downward trend the drink consumed quickly within 60 min. This results are consistent with the previous study about ethanol concentrations in exhaled breath^41^.

### Mechanism of the Gas Sensor

Based on the above analysis of gas sensing characteristic, the sensing mechanism of both In_2_O_3_/Au and In_2_O_3_ based sensors could be modeled using the surface–controlled model^42^,^43^. Generally, when the In_2_O_3_ sensor is exposed to air, it interacts with oxygen by transferring electrons from the conduction band to the adsorbed oxygen atoms, forming reactive oxygen species (O_2_ˉ, O^2^ˉ, and Oˉ). The reaction can be expressed as:
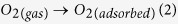

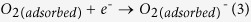

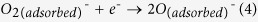

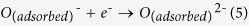


Based on the above reactions, a depletion layer is created due to the consumption of the electron in the surface region of the In_2_O_3_ sensor. This leads to the increase of the resistance in the In_2_O_3_ sensor. When the In_2_O_3_ sensor is placed into the reducing gas (acetone or ethanol in our case), the reactive oxygen ions will react with the analysis gas molecules and then the electrons are released back to the In_2_O_3_ sensor, resulting in the thinning of the depletion layer and the decrease of the resistance of the In_2_O_3_ sensor^44^. The above explanation can be expressed by the following equations:
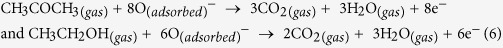


### Obviously, the sensor performance will be strongly dependent of the amount of ion sorption of oxygen

In general, at low temperature, active sites at the semiconductor surface are rare and the amount of ion sorption of oxygen is deficient. These can lead to incompletely reaction of analysis gas. So the response is poor at low temperature. With increasing the working temperature, the ion sorption of oxygen on the sensor surface quickly increases, and the adsorbed oxygen gives rise to Schottky potential barriers at grain boundaries, and thus increases the resistance of the sensor surface. When reducing gases are introduced, the response will increase. While, further increasing the working temperature, the charge-carrier concentration and the conductivity increase and the Debye length decreases, which leads to the decrease of response. In fact, the response process of a semiconductor sensor on temperature is very complex. Its effect factors include the rates of adsorption and desorption of oxygen and reducing gases, the rate of surface decomposition of reducing gases, the charge carrier concentration, and the Debye length in the semiconductor^45^. Mutual effect makes different gases have characteristic optimum oxidation temperatures. Thus, the as studied gas sensors, especially S3 In_2_O_3_/Au NRs, exhibits good dual mode sensing properties both to acetone and ethanol gases in this work.

When Au are introduced, the uniform distributed Au in In_2_O_3_/Au NRs could promote the reactive oxygen species as chemical sensitizer and the most probable mechanism leading to improve sensor response is chemical sensitization via spillover effect^23^,^46^,^47^. Ionsorptions of oxygen ions can occur on the Au NPs surface due to the highly conductive nature and availability of free electrons in gold. Then the as created activated oxygen species are spilled onto the metal oxides surface and interact with the absorbed oxygen. This consists with the increasing deficient oxygen atomic ratio percentages after introducing of Au, as studied in XPS (Fig. 4b). This process has been considered to result in greater and faster reaction between analytic molecules and adsorbed oxygen in a certain range (as shown in Fig. 5 and Table S1). Note that the oxygen molecules can briefly diffuse to a catalyst metal NPs before it has an opportunity to be absorbed^48^. Thus, this “chemical sensitization” mechanism is considered to be the predominant mechanism responsible for enhancement of the gas sensing properties by adding the Au NPs. Further the XPS binding energy peak positions also proved the existence of the Au/In_2_O_3_ interaction. The shift of the In 3d peak to the higher binding energy is attributed to the interaction of In_2_O_3_ with Au and then leads to a modification of the surface electronic structure. This brings out the second role of Au as modulator of the surface electronic structure. So in our studies, the response value and response dynamic process are gradually increased with the increasing of the amount of Au at first (pure In_2_O_3_, S4, and S5). When Au amount further increases, the dynamic process slows down, this may attributed to the change of corresponding optimal working temperature.

Generally, at low temperature most semiconductor metal oxides gas sensor materials are insulators rather than semiconductor, thus the gas sensing sensitivity is very poor. After the introduction of Au, ionsorption of oxygen ions can occur on the metal NPs surface at a low temperature (even at room temperature) due to the highly conductive nature, availability of free electrons, and chemical sensitization in Au^48^. That is the reason that the optimal working temperature are gradually decreased by increasing the amount of the Au (Fig. 5), and this effect is more obvious when the introduced amount of Au is relatively large (atom ratio of Au/In > 3.6% in our case). As is known, the dynamic process slow down with the decrease of working temperature due to the Eqs (2)–(6) are related to the thermodynamics. In our case, when the introduced Au amount is low, it has little influence on the optimal working temperature. When the Au amount further increases, the optimal working temperature decreases, however, because more ionsorption of oxygen ions can occur on the Au NPs surface, the corresponding response values can have little change (ethanol gas) or even increasing (acetone gas). However, because of the decrease of working temperature, the response time and recovery time become longer.

However, despite the semiconductor oxide based gas sensors exhibited many advantage which meet the criteria of an ideal breath sensor, such as high response to low concentrations of VOCs, fast dynamic process, low-cost, simple and inexpensive to disposable^13^, it is still a long way to apply these sensors on breath analysis in clinical diagnose. How to avoid the effect of humidity and improve the accuracy of specific VOCs detection against other interference specie needs further study. Since one disease may corresponding to more than one biomarkers in exhaled breath^14^, following the idea of this work, the dual mode detection may help to increase the accuracy of VOCs detection in semiconductor oxide based gas sensors.

## Conclusions

In summary, In_2_O_3_/Au NRs as well as pure In_2_O_3_ were fabricated using a facile co–precipitation technique. It is believed that the initial In(NO_3_)_3_ amount and reaction time have little effect on the morphology of In_2_O_3_/Au NR and the Au NPs were uniformly dispersed in In_2_O_3_/Au NRs. The dual mode gas sensing properties of different In_2_O_3_/Au NRs sensors compared with pure In_2_O_3_ were carefully studied, especially for very low concentration of acetone and ethanol. The results demonstrate that the optimal S3 In_2_O_3_/Au NRs sensor can effectively detect acetone at 250 °C and ethanol at 400 °C, which has response of 2.8 to 1 ppm acetone, and 9.8 to 5 ppm ethanol and low actual detection limit of 0.1 ppm to acetone and 0.05 ppm to ethanol. Besides, a humidity compensation method is applied to increase the accuracy of the sensor response in high humidity environment. Moreover, clinic tests were also performed, indicating that the In_2_O_3_/Au NRs sensor has the ability to actual detection acetone and ethanol biomarkers in exhaled breath. The present work suggests that the “spillover effects” of introduced Au NPs increased the activity oxygen species of In_2_O_3_ NRs and endowed In_2_O_3_/Au NRs gas sensor with enhanced dual mode gas sensing properties.

## Materials and methods

### Synthesis of Au NRs

All the experiment reagents were used as received and without further purification. The Au NRs were prepared following the seed–mediated growth method reported by EI–Sayed and co–workers^49^. Brief descriptions were as follows: 1) Preparation of the seed solution. 2.5 mL of HAuCl_4_ (1 mM aqueous) and 7.4 mL of cetyltrimethylammonium bromide (CTAB, 0.1 M aqueous) were mixed together, followed by the injection of 0.6 mL freshly prepared NaBH_4_ (10 mM aqueous) solution and magnetic stirring at 28 °C for 2 h. 2) Preparation of the Au NRs. In a flask, 13.2 mL of CTAB (0.1 M aqueous) was mixed with 12 mL of HAuCl_4_ (1 mM aqueous), and then 240 μL of AgNO_3_ (10 mM aqueous) and 220 μL of HCl (2 M aqueous) were added. After mixing, 192 μL of ascorbic acid (AA, 0.1 M) was added. While continuously stirring this mixture, 120 μL of the as–synthesized seed solution was added to initiate the growth of Au NRs. Then, these NRs were kept at 28 °C for 5 h to ensure full growth. Finally, after centrifuging at 13 000 rpm for 15 min, the upper liquid containing excess CTAB was removed, and NRs were left over in 40 mL deionized water.

### Preparation of In_2_O_3_/Au NRs samples

The In_2_O_3_/Au NRs were synthesized through co–precipitation method and annealed. First, various amounts (0.1 g, 0.15 g, 0.2 g and 0.3 g) of In(NO_3_)_3_ were dissolved in 10 mL of deionized water which contained 1.8 g urea. After stirring, 20 mL of the as–synthesized Au NRs aqueous solution as motioned above was added. Then, the mixed solution heated to 80 °C and kept refluxing for 2 h in the oil bath. After self–cooling down to room temperature, the reaction product was separated by centrifugation and washing with deionized water for three times, then further dried at 60 °C for 6 h. At this time, the precursor NRs of [Au@In(OH)CO_3_] was obtained. Finally, the as–synthesized products were annealed in a tube furnace with a rising rate of 2.5 °C/min from room temperature to 600 °C and maintained for 3 h at 600 °C to obtain the In_2_O_3_/Au NRs samples. For comparison, pure In_2_O_3_ was also synthesized as follows, 0.2 g In(NO_3_)_3_ was dissolved in 30 mL of deionized water solution containing 1.8 g urea and no Au NRs were added, and the other processes were just the same with the above.

### Characterization

The morphology of the samples was inspected using JEOL JSM–7500F field emission SEM (Japan) with accelerating voltage of 15 kV and gold sputtering on surface. TEM and high resolution TEM (HRTEM) images were recorded on JEM–2010 transmission electron microscope under a working voltage of 200 kV. The phase structure of the samples were characterized by XRD; XRD patterns were conducted on Rigaku D/max 2550 using a monochromatized Cu target radiation resource (λ = 1.5045 Å) and the corresponding lattice constants of the samples were calculated by MDI Jade 5.0 software. XPS were conducted on an ESCAlab250 Analytical XPL Spectrometer with a monochromatic Al KR source. All the binding energies were referred to the C1s peak at 284.7 eV of the surface adventitious carbon. The fitted peaks in XPS spectra were deconvoluted using the XPS Peak 4.1 software.

### Fabrication and measurement of gas sensing properties

The as–synthesized samples were mixed with ethanol in the weight ratio of 5:1 to form a paste. The paste was coated on a ceramic tube on which a pair of gold electrodes was previously printed. A small spring–like Ni–Cr alloy was inserted into the ceramic tube to provide the operating temperature. After the solvent was evaporated, the ceramic tube with samples thin layer was sintered in an oven for 2 h at 400 °C. After sintering, the gas sensors were thermal aged with a heating voltage of 5 V at the ageing equipment for 6 days before the first measurement.

The gas–sensing properties were measured on a WS–30A system (Weisheng Instruments Co., Zhengzhou, China) and determined under laboratory conditions (25 ± 1RH%, 26 ± 2 °C).The response is defined as S = R_a_/R_g_ (R_a_ and R_g_ are the resistance for sensors in air and in target gas, respectively.). The response and recovery times are defined as the time required reaching 80% of the final equilibrium value, and the details of the measurement are similar to those reported in the literature^50^. To clinic test, the sampling gas were collected in an air pocket (2L).

## Additional Information

**How to cite this article**: Xing, R. *et al*. Preparation and Gas Sensing Properties of In_2_O_3_/Au Nanorods for Detection of Volatile Organic Compounds in Exhaled Breath. *Sci. Rep*. **5**, 10717; doi: 10.1038/srep10717 (2015).

## Supplementary Material

Supplementary Information

## References

[b1] Deng C. H., Zhang J., Yu X. F., Zhang W. & Zhang X. M. Determination of acetone in human breath by gas chromatography-mass spectrometry and solid-phase microextraction with on-fiber derivatization. J. Chromatogr. B 810, 269–275 (2004). 10.1016/j.jchromb.2004.08.013 15380724

[b2] Peng G. . Diagnosing lung cancer in exhaled breath using gold nanoparticles. Nat. Nanotechnol. 4, 669–673 (2009). 19809459 10.1038/nnano.2009.235

[b3] Horvath I. . Exhaled breath condensate: methodological recommendations and unresolved questions. Eur. Respir. J. 26, 523–548 (2005). 16135737 10.1183/09031936.05.00029705

[b4] Itoh T., Nakashima T., Akamatsu T., Izu N. & Shin W. Nonanal gas sensing properties of platinum, palladium, and gold-loaded tin oxide VOCs sensors. Sensors Actuat. B-Chem. 187, 135–141 (2013).

[b5] Haick H., Broza Y. Y., Mochalski P., Ruzsanyi V. & Amann A. Assessment, origin, and implementation of breath colatile cancer markers. Chem. Soc. Rev. 43, 1423–1449 (2014). 24305596 10.1039/c3cs60329f PMC4909138

[b6] Kamble V. B. & Umarji A. M. Gas sensing response analysis of p-type porous chromium oxide thin films. J. Mater. Chem. C 1, 8167–8176 (2013).

[b7] Luo J. . Nanoparticle-structured thin film sensor arrays for breath sensing. Sensors Actuat. B-Chem. 161, 845–854 (2012).

[b8] Jones A. W. & Andersson L. Comparison of ethanol concentrations in venous blood and end-expired breath during a controlled drinking study. Forensic Sci. Int. 132, 18–25 (2003). 12689747 10.1016/s0379-0738(02)00417-6

[b9] Gullberg R. G. Breath alcohol measurement variability associated with different instrumentation and protocols. Forensic Sci. Int. 131, 30–35 (2003). 12505468 10.1016/s0379-0738(02)00375-4

[b10] Ryabtsev S. V., Shaposhnick A. V., Lukin A. N. & Domashevskaya E. P. Application of semiconductor gas sensors for medical diagnostics. Sensors Actuat. B-Chem. 59, 26–29 (1999).

[b11] Teshima N., Li J. Z., Toda K. & Dasgupta P. K. Determination of scetone in breath. Anal. Chim. Acta 535, 189–199 (2005).

[b12] Shin J. . Thin-wall assembled SnO_2_ fibers functionalized by catalytic Pt nanoparticles and their superior exhaled-breath-sensing properties for the diagnosis of diabetes. Adv. Funct. Mater. 23, 2357–2367 (2013).

[b13] Konvalina G. & Haick H. Sensors for Breath Testing: From Nanomaterials to Comprehensive Disease Detection. Accounts Of Chemical Research 47, 66–76, (2014). 23926883 10.1021/ar400070m

[b14] Broza Y. Y. & Haick H. Nanomaterial-based sensors for detection of disease by volatile organic compounds. Nanomedicine 8, 785–806, (2013). 23656265 10.2217/nnm.13.64

[b15] Tisch U. & Haick H. Nanomaterials for cross-reactive sensor arrays. Mrs Bulletin 35, 797–803, (2010).

[b16] Righettoni M., Tricoli A. & Pratsinis S. E. Si:WO_3_ sensors for highly selective detection of acetone for easy diagnosis of diabetes by breath analysis. Anal. Chem. 82, 3581–3587 (2010). 20380475 10.1021/ac902695n

[b17] Inyawilert K. . Ultra-rapid VOCs sensors based on sparked-In_2_O_3_ sensing films. Sensors Actuat. B-Chem. 192, 745–754 (2014).

[b18] Liu C., Hayashi K. & Toko K. Au nanoparticles decorated polyaniline nanofiber sensor for detecting volatile sulfur compounds in expired breath. Sensors Actuat. B-Chem. 161, 504–509 (2012).

[b19] Wang Y. L., Jiang X. C. & Xia Y. N. A solution-phase, precursor route to polycrystalline SnO_2_ nanowires that can be esed for gas sensing under ambient conditions. J. Am. Chem. Soc. 125, 16176–16177 (2003). 14692744 10.1021/ja037743f

[b20] Paska Y., Stelzner T., Christiansen S. & Haick H. Enhanced sensing of nonpolar volatile organic compounds by silicon nanowire field effect transistors. Acs Nano 5, 5620–5626 (2011). 21648442 10.1021/nn201184c

[b21] Xu Y. . Detection and identification of breast cancer volatile organic compounds biomarkers using highly-sensitive single nanowire array on a chip. J. Biomed. Nanotechnol. 9, 1164–1172 (2013). 23909130 10.1166/jbn.2013.1651

[b22] Wang Y. . Brookite TiO_2_ decorated alpha-Fe_2_O_3_ nanoheterostructures with rod morphologies for gas sensor application. J. Mater. Chem. A 2, 7935–7943 (2014).

[b23] Choi K. J. & Jang H. W. One-dimensional oxide nanostructures as gas-sensing materials: review and issues. Sensors 10, 4083–4099 (2010). 22319343 10.3390/s100404083 PMC3274262

[b24] Jiang T. . Synergic effect within n-type inorganic-p-type organic nano-hybrids in gas sensors. J. Mater. Chem. C 1, 3017–3025 (2013).

[b25] Chen J., Xu L. N., Li W. Y. & Gou X. L. alpha-Fe_2_O_3_ nanotubes in gas sensor and lithium-ion battery applications. Adv. Mater. 17, 582–586 (2005).

[b26] Chen J., Li J., Li J., Xiao G. & Yang X. Large-scale syntheses of uniform ZnO nanorods and ethanol gas sensors application. J. Alloy Compd. 509, 740–743 (2011).

[b27] Liu X. . 3D hierarchically porous ZnO structures and their functionalization by Au nanoparticles for gas sensors. J. Am. Chem. 21, 349–356 (2011).

[b28] Wang C., Chen D. & Jiao X. Flower-like In_2_O_3_ nanostructures derived from novel precursor: synthesis, characterization, and formation mechanism. J. Phys. Chem. C 113, 7714–7718 (2009).

[b29] Liu T. . Yb_2_O_3_/Au upconversion nanocomposites with broad-band excitation for solar cells. J. Phys. Chem. C 118, 3258–3265 (2014).

[b30] Yu D. B. . Metastable hexagonal In_2_O_3_ nanofibers templated from InOOH nanofibers under ambient pressure. Adv. Funct. Mater. 13, 497–501 (2003).

[b31] Chen C., Chen D., Jiao X. & Chen S. In_2_O_3_ nanocrystals with a tunable size in the range of 4-10 nm: one-step synthesis, characterization, and optical properties. J. Phys. Chem. C 111, 18039–18043 (2007).

[b32] Bera P. & Hegde M. S. Characterization and catalytic properties of combustion synthesized Au/CeO_2_ catalyst. Catal. Lett. 79, 75–81 (2002).

[b33] Si R. & Flytzani-Stephanopoulos M. Shape and crystal-plane effects of nanoscale ceria on the activity of Au-CeO_2_ catalysts for the water-gas shift reaction. Angew. Chem. Int. Edit. 47, 2884–2887 (2008). 10.1002/anie.200705828 18327859

[b34] Huo Z., Tsung C.-k., Huang W., Zhang X. & Yang P. Sub-two Nanometer Single Crystal Au Nanowires. Nano Lett. 8, 2041–2044 (2008). 18537294 10.1021/nl8013549

[b35] Mitsubayashi K., Matsunaga H., Nishio G., Toda S. & Nakanishi Y. Bioelectronic sniffers for ethanol and acetaldehyde in breath air after drinking. Biosens. Bioelectron. 20, 1573–1579 (2005). 15626611 10.1016/j.bios.2004.08.007

[b36] Xu L. . Electrospinning preparation and room temperature gas sensing properties of porous In_2_O_3_ nanotubes and nanowires. Sens. Actuators B-Chem. 147, 531–538, (2010).

[b37] Kato S., Burke P. J., Koch T. H. & Bierbaum V. M. Formaldehyde in human cancer cells: detection by preconcentration-chemical ionization mass spectrometry. Anal. Chem. 73, 2992–2997 (2001). 11467545 10.1021/ac001498q

[b38] Jiang H. & Zou J. Identification of new stilbenoids-formaldehyde ddducts by isotope labeling and electrospray ionization mass spectrometry. Eur. Food Res. Technol. 236, 425–434 (2013).

[b39] Dovgolevsky E., Konvalina G., Tisch U. & Haick H. Mono layer-Capped Cubic Platinum Nanoparticles for Sensing Nonpolar Analytes in Highly Humid Atmospheres. J. Phys. Chem. C 114, 14042–14049 (2010).

[b40] Konvalina G. & Haick H. Effect of Humidity on Nanoparticle-Based Chemiresistors: A Comparison between Synthetic and Real-World Samples. ACS Appl. Mater. Interfaces 4, 317–325, (2012). 22121824 10.1021/am2013695

[b41] Jones A.W. & Andersson L. Comparison of ethanol concentrations in venous blood and end-expired breath during a controlled drinking study. Forensic Science International 132, 18–25 (2003). 12689747 10.1016/s0379-0738(02)00417-6

[b42] Mattei G. . Cookie-like Au/NiO nanoparticles with optical gas-sensing properties. Adv. Mater. 19, 561–564 (2007).

[b43] Feng P., Wan Q. & Wang T. H. Contact-controlled sensing properties of flowerlike ZnO nanostructures. Appl. Phys. Lett. 87, 213111-1-3 (2005).

[b44] Kim J., Kim W. & Yong K. CuO/ZnO heterostructured nanorods: photochemical synthesis and the mechanism of H_2_S Gas Sensing. J. Phys. Chem. C 116, 15682–15691 (2012).

[b45] Lee A. P. & Reedy B. J. Temperature modulation in semiconductor gas sensing. Sens. Actuators B-Chem. 60, 35–42 (1999).

[b46] Xiang Q. . Au nanoparticle modified WO_3_ nanorods with their enhanced properties for photocatalysis and gas sensing. J. Phys. Chem. C 114, 2049–2055 (2010).

[b47] Du N., Zhang H., Ma X. & Yang D. Homogeneous coating of Au and SnO_2_ nanocrystals on carbon nanotubes via layer-by-layer assembly: a new ternary hybrid for a room-temperature CO gas sensor. Chem. Commun. 48, 6182–6184 (2008). 10.1039/b812695j 19082113

[b48] Kolmakov A., Klenov D. O., Lilach Y., Stemmer S. & Moskovits M. Enhanced gas sensing by individual SnO2 nanowires and nanobelts functionalized with Pd catalyst particles. Nano Lett. 5, 667–673 (2005). 15826106 10.1021/nl050082v

[b49] Huang X. H., El-Sayed I. H., Qian W. & El-Sayed M. A. Cancer cell imaging and photothermal therapy in the near-infrared region by using gold nanorods. J. Am. Chem. Soc. 128, 2115–2120 (2006). 16464114 10.1021/ja057254a

[b50] Xing R. Q. . Three-dimensional ordered SnO_2_ inverse opals for superior formaldehyde gas-sensing performance. Sensors Actuat. B-Chem. 188, 235–241 (2013).

