# Supplementary material for: Preparation and Gas Sensing Properties of In2O3/Au Nanorods for Detection of Volatile Organic Compounds in Exhaled Breath
Source: Sci Rep. 2015 Jun 1;5:10717. doi: 10.1038/srep10717 (PMC5377237; doi:10.1038/srep10717)
Supplement: Supplementary Information [file srep10717-s1.pdf]

Preparation and Gas Sensing Properties of In_2O_3/Au Nanorods for Detection of Volatile Organic Compounds in Exhaled Breath

Ruiqing Xing,[a] Lin Xu,[a] * Jian Song,[a] Chunyang Zhou,[a] Qingling Li,[a] Dali Liu,[a] Hongwei Song*[a,b]

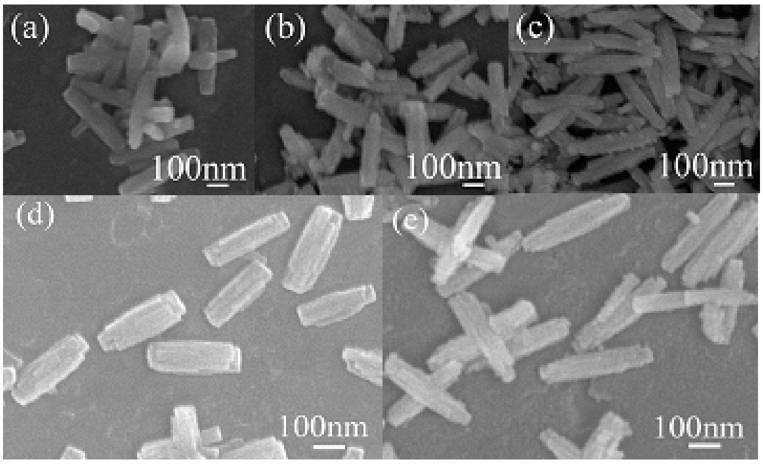

Figure 1S

Figure S1 SEM images of different In_2O_3/Au NRs with (a) 0.1 g (b) 0.15 g and (c) 0.3 g amount of initial $In(NO_3)_3$ at the reaction time of 120 min, (d-e) SEM images of precursor In_2O_3/Au NRs with 0.2 g initial $In(NO_3)_3$ at the reaction time of 50 min and 180 min.

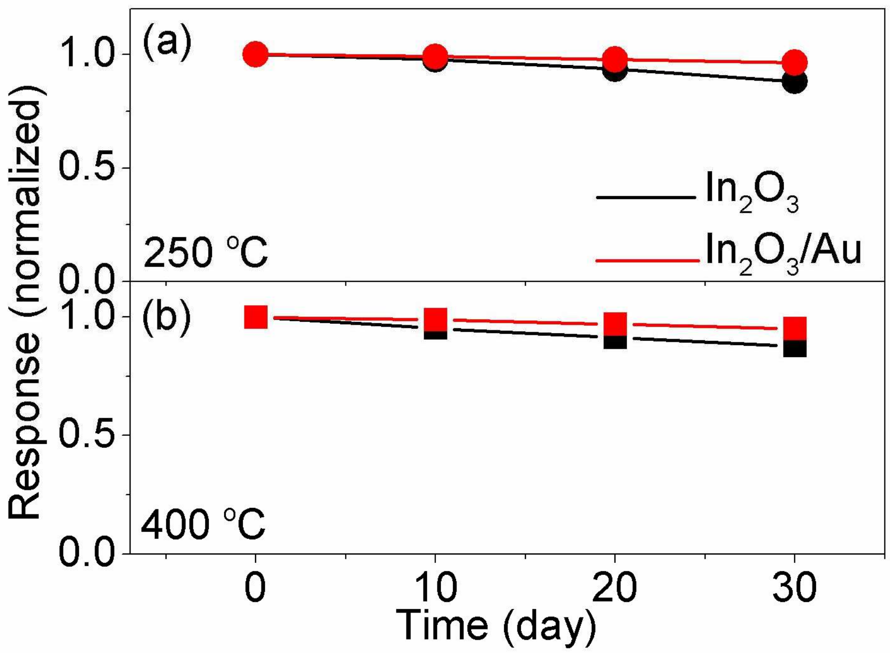

Figure S2 The long–term stabilities of S3 In_2O_3/Au NRs compared with pure In_2O_3 gas sensors to 1 ppm acetone and 5 ppm ethanol.

Table S1 The response times and recovery times of different sensors at corresponding optimal temperature. (Acetone is 2 ppm and ethanol is 10 ppm)

~ Time (s)		S1	S2	S3	S4	In_2O_3
acetone	Res.	13	9	10	9	15
	Rec.	84	63	20	25	25
ethanol	Res.	7	10	7	7	8
	Rec.	39	23	14	15	16

Table S2. Response times and recovery times of pure In_2O_3 and S3 In_2O_3/Au NRs gas sensors *vs.* different acetone (at 250°C) and ethanol (at 400°C) concentration.

~Time (s)	ppm	Acetone concentration					Ethanol concentration				
		0.2	1	2	10	20	2	5	10	50	100
In_2O_3	Res.	15	14	15	14	16	11	8	8	9	11
	Rec.	28	25	25	25	28	16	18	16	18	21
In_2O_3/Au	Res.	10	12	10	9	13	10	8	7	9	11
	Rec.	18	17	20	17	20	10	16	14	13	14
